# Sterigmatocystin Limits *Plasmodium falciparum* Proliferation and Transmission

**DOI:** 10.3390/ph14121238

**Published:** 2021-11-29

**Authors:** Guodong Niu, Komal Kalani, Xiaohong Wang, Jun Li

**Affiliations:** 1 Department of Biological Sciences, Florida International University, Miami, FL 33199, USA; gniu@fiu.edu (G.N.); kkalani@fiu.edu (K.K.); xiawang@fiu.edu (X.W.); 2 Biomolecular Sciences Institute, Florida International University, Miami, FL 33199, USA

**Keywords:** *Penicillium janthinellum*, antimalarial agents, fungal metabolites, natural products

## Abstract

As part of our drug discovery program against malaria, the *Penicillium janthinellum* extract was discovered to inhibit *P. falciparum* proliferation in blood and transmission to mosquitoes. Bioactivity-guided fractionation of *P. janthinellum* extraction was carried out using chromatographic techniques. We determined the activities of fractions against *Plasmodium falciparum* asexual stage parasite proliferation in culture and sexual stage parasite transmission to mosquitoes using standard membrane feeding assays (SMFA). One active compound was isolated. Based on mass spectrometry and nuclear magnetic resonance profiles, the compound was structurally determined to be sterigmatocystin. Sterigmatocystin inhibited *P. falciparum* proliferation in the blood with an IC_50_ of 34 µM and limited the sexual parasites to infect mosquitoes with an IC_50_ of 48 µM. Meanwhile, sterigmatocystin did not show any acute toxicity to human kidney cells at a concentration of 64 µM or lower. Sterigmatocystin can be used as a drug lead for malaria control and as a probe to understand molecular mechanisms of malaria transmission.

## 1. Introduction

Malaria is one of the most severe mosquito-transmitted diseases, and it caused 229 million cases of new malaria infections in 2019 and led to 409,000 deaths worldwide [1]. The malaria cases and deaths mainly occurred in tropical and subtropical regions, particularly in sub-Saharan Africa and Southeast Asia [2]. The wide spread of drug-resistant *Plasmodium* parasites urges the development of novel antimalarial strategies [3].

Fungi produce structurally novel and diverse secondary metabolites as potential drug leads against many diseases [4]. Fungal metabolites also remain a consistent source of antimalarial compounds, and a great structure-diversity of compounds was discovered with potential anti-*Plasmodium* activity [5]. For example, a series of macrolides, which contain a large macrocyclic lactone ring with one or more deoxy sugars, isolated from several fungal species showed various antimalarial effects [6]. Another huge class of naturally occurring hydrocarbons consisting of multiple isoprene units is terpene metabolites, which were reported to show antimalarial activities. Among them, two trichothecenes-isolated *Myrothecium verrucaria* showed potent antimalarial activity with an IC_50_ value below 20 nM [7]. In addition, peptides, such as cyclohexadepsipeptide Pullularins A and B, isolated from the fungus *Aureobasidium pullulans*, exhibited antimalarial activities with IC_50_ values of 4.6 and 4.2 μM, respectively [8]. Other fungi-derived classes of compounds with antimalaria activity include piperazine derivatives, tetrameric acid derivatives, xanthones, pyridine, coumarins, quinone derivatives, hydronaphthalenone derivatives, and tryptophan derivatives.

By using an established high-throughput screening platform, we discovered several fungal metabolites that were active against *P. falciparum* parasites in the mosquito stage [9]. *P*-orlandin, isolated from *Aspergillus niger*, was able to significantly reduce *P. falciparum* infection in mosquitoes significantly, and thus has the potential to block malaria transmission [9]. Another noteworthy compound, asperaculane B, isolated from *Aspergillus aculeatus*, is a new dual-functional antimalarial lead that is active against both the asexual stage and sexual stage of *P. falciparum*. It can be developed as a new type of malaria transmission-blocking agent [10]. Most recently, a novel isolated compound, pulixin from *Purpureocillium lilacinum*, was reported to block the transmission of the parasite to mosquitoes with an IC_50_ of 11 µM and inhibit the proliferation of asexual-stage *P. falciparum* with an IC_50_ of 47 nM [11].

Penicillin from *Penicillium* mold is known as the first fungal metabolite, and its discovery started the era of bioactive compounds isolated from fungus resources. *Penicillium* species are some of the most common fungi observed worldwide and are known for their diverse fungal metabolites. Several bioactive compounds were isolated and characterized from these species, and many more bioactive compounds need to be separated and identified based on their wider therapeutic usage. Recently, a promising antimalarial activity was reported in *Penicillium janthinellum* against chloroquine-resistant *P. falciparum* INDO (*PfINDO*) strain at its asexual stage along with less cytotoxicity against HEK239T cells. However, no noted compound has been isolated from this fungus so far to target malaria [12]. A screening using the recently established global fungal extract library [13] also found a crude extract from *P. janthinellum* that inhibited malaria transmission. This prompted us to carry out the bioactivity-guided isolation and identification of antimalarial compounds from *P. janthinellum*.

## 2. Results

### 2.1. Isolation of Active Compounds from the Candidate Fungal Extract

**Figure 1:**
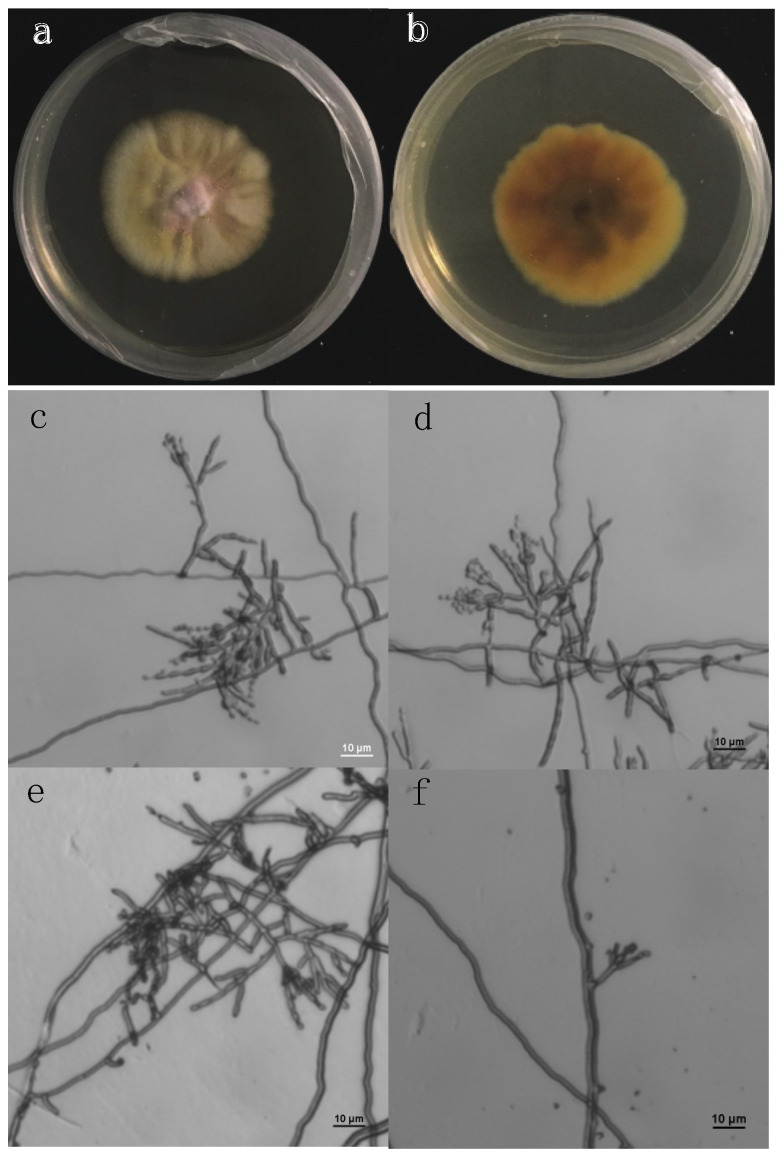
Fungal colonies and spores. (**a**,**b**): front and back view of fungal colony on a culture plate. (**c**–**f**): morphology of fungal spores.

The fungus of *P. janthinellum* was the isolate of GFEL-26F6 in our recently constructed global fungal library [13]. The fungal colony is yellow with pink in the center on an MEA agar plate after 2-week incubation at room temperature (Figure 1a). Under the microscope, the fungus showed a brush-like sporulating structure, producing long green chains of dusty, single-celled spores (Figure 1c–f) [12,14,15]. We PCR-amplified ITS sequence from this fungus, blasted the sequence against nucleotide collection in NCBI, and found it 99.7% identical to *P. janthinellum*. This ITS sequence was deposited into NCBI with an accessible number of GU565141.1.

**Figure 2:**
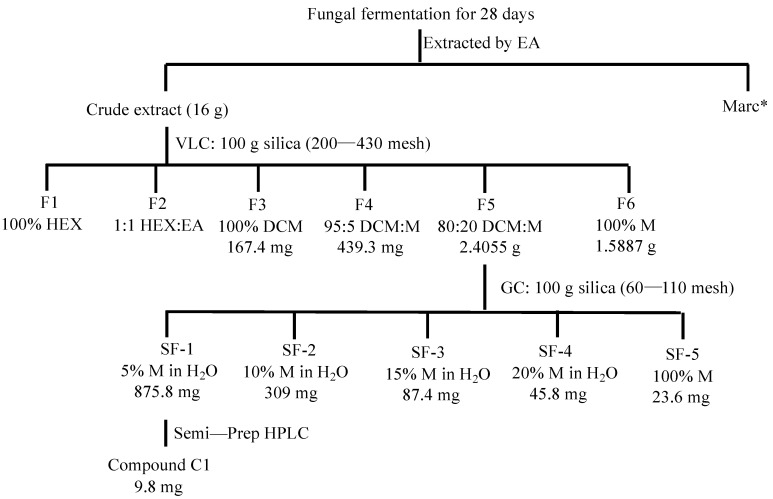
Schematic presentation of isolation of pure compounds from *P.* *janthinellum*. Marc*: residue incubated and discarded after extraction with ethyl acetate. VLC: Vacuum Liquid Chromatography; GC: Gravity column; EA: ethyl acetate; HEX: hexane; DCM: dichloromethane; M: methanol.

The crude extract was tested for its inhibition activity against asexual stage *P. falciparum*. Results showed that 30 µg/mL crude extract was able to completely inhibit asexual stage *P. falciparum* proliferation (Table 1). The fungal extract was fractioned different matrix and solvents as shown in Figure 2. The inhibition activities of these fractions against asexual stage *P. falciparum* were examined. Results showed that different fractions had different activities, and 30 µg/mL fraction 5 (F5) from crude extract was able to inhibit parasite proliferation (Table 1) completely. Therefore, F5 was further chromatographed over a silica gel gravity column and sequentially eluted different concentration of methanol in water. The subfractions were named SF-1 to SF-5, respectively, and their activities against asexual stage parasites were determined. The results showed 30 µg/mL SF-1 completely inhibited *P. falciparum* proliferation in blood (Table 1).

**Figure 3:**
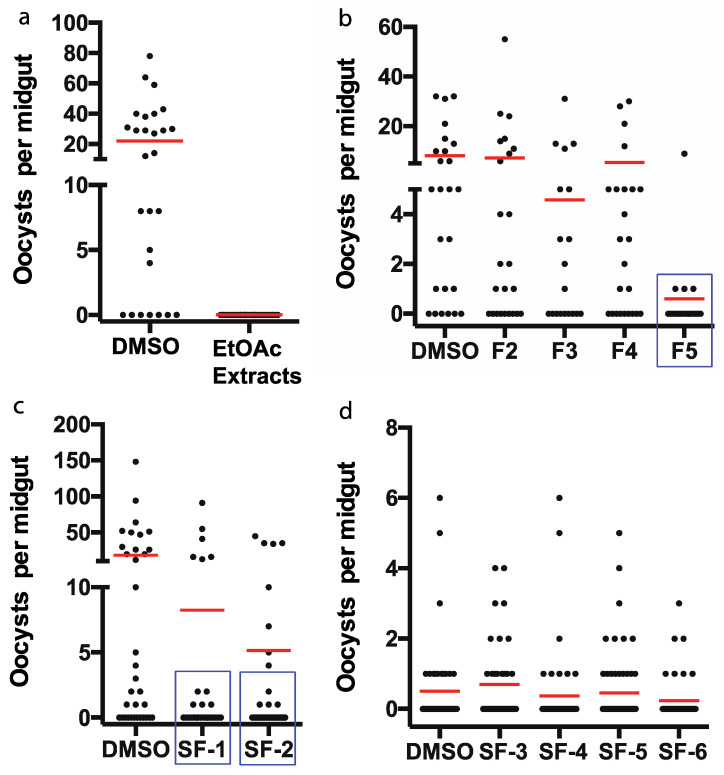
Inhibition of chromatography fractions. (**a**,**b**) or subfractions (**c**,**d**) of fungal extract on *P. falciparum* infection in *An. gambiae* was analyzed by SMFA. (**a**,**b**) activities of crude extract fractions through silica gel liquid chromatography. (**c**,**d**) activities of subfractions from active fraction F5 by a silica gel gravity column. concentration of samples is 30 µg/mL. *p*-value between control (DMSO) and a sample was calculated by Mann-Whitney test. Samples, highlighted by blue rectangles, significantly inhibited *P. falciparum* transmission to mosquitoes (*p* < 0.05). Each experiment was conducted twice, and similar results were obtained.

Furthermore, we analyzed these fractions for their activities against sexual stage *P. falciparum* transmission to *An. gambiae* using SMFA. Results showed that 30 µg/mL crude extract, F5, SF-1, or SF-2, had significant inhibition on parasite transmission to mosquitoes (*p* < 0.05), leading to remarkable fewer oocysts (Figure 3).

**Figure 4:**
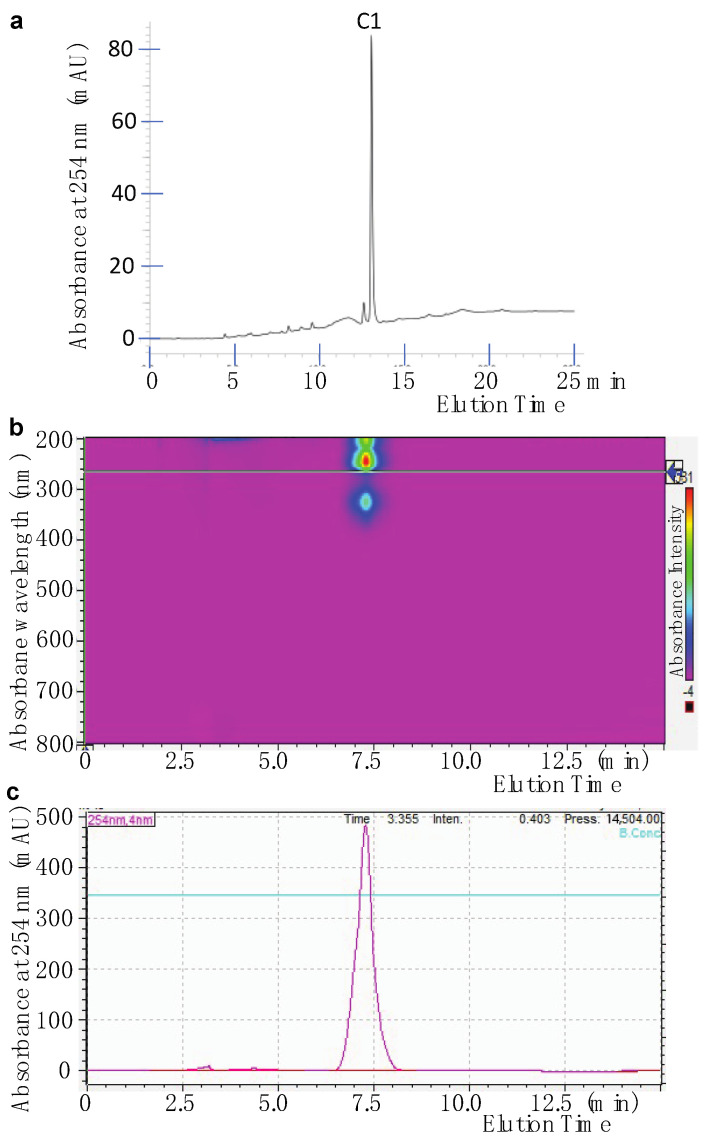
Separation of an active compound by HPLC. (**a**) HPLC profile of active fraction SF-1 containing active compound C1. (**b**) Analysis of purity of C1 using HPLC showed one major peak at 7.4 min by A_200–800_. (**c**) A_254_ profile of elution from HPLC.

Finally, the active fraction SF-1 was further purified on semi-preparative HPLC using a gradient solvent of methanol/water. One major compound (C1) was isolated from SF-1 (Figure 4a). The purity of C1 was further verified using the HPLC with a different solvent. One band was detected by a UV-Vis detector (Figure 4b). Based on the area of the peaks at A_254_ (Figure 4c), the compound C1 was >99% in purity.

### 2.2. Identification of the Purified Compound

**Figure 5:**
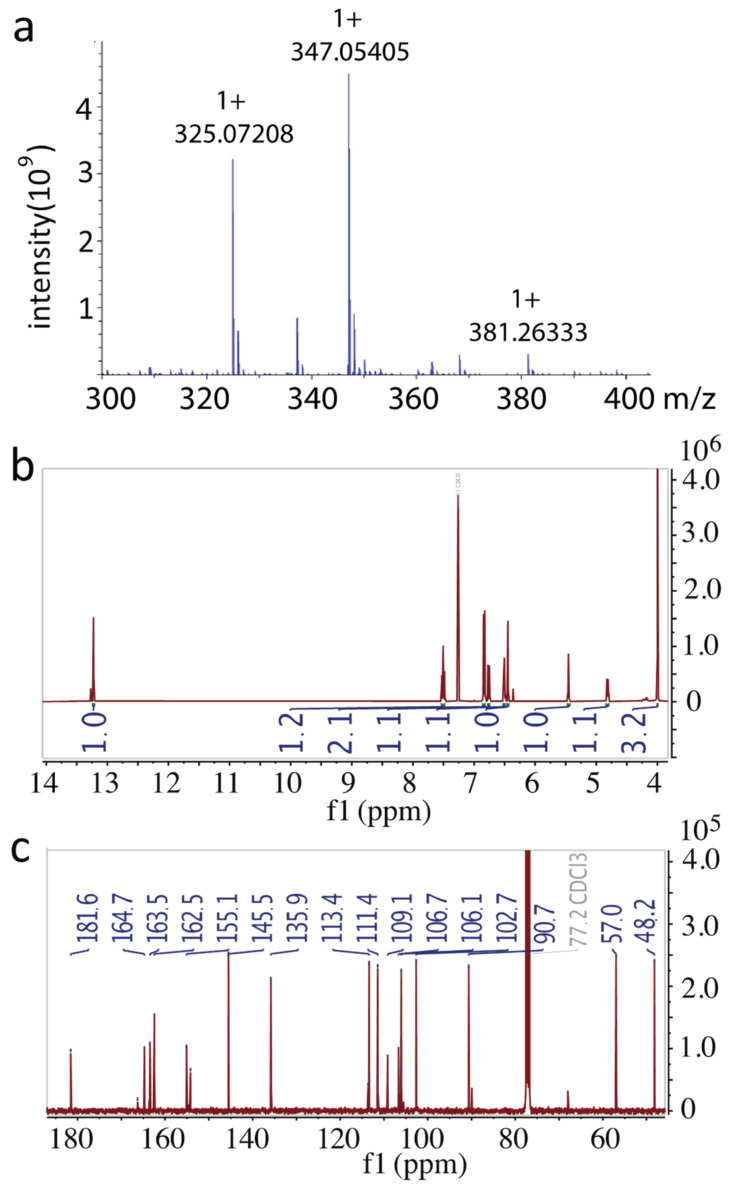
Structure identification of active compound. (**a**) Positive ion mode mass spectrum that showed 325.07208 [M + H]^+^ and 347.05405 [M + Na]^+^. (**b**) ^1^H NMR spectrum. (**c**) ^13^C NMR spectrum.

Next, we analyzed that the structure of compound C1. The positive mode electrospray ionization mass spectrometry (ESIMS) was used to analyze precise mass. Two major peaks were observed at *m*/*z* 325.07208 [M + H]^+^ and 347.05405 [M + Na]^+^ (Figure 5a). Therefore, this compound possessed a molecular weight of 324.06423 Da. Next, ^1^H and ^13^C NMR of this pure compound in chloroform-d were performed. The proton chemical shifts of [δ_H_] (Figure 5b) and carbon chemical shifts [δ_C_] (Figure 5c) were identical to sterigmatocystin, a fungal metabolite released from some *Penicillium* fungal species [15]. The chemical shifts of the pure compound were summarized in Table 2. Based on the NMR data and the accurate MS data, we determined compound C1 to be sterigmatocystin.

### 2.3. The Antimalarial Properties of the Pure Compound

**Figure 6:**
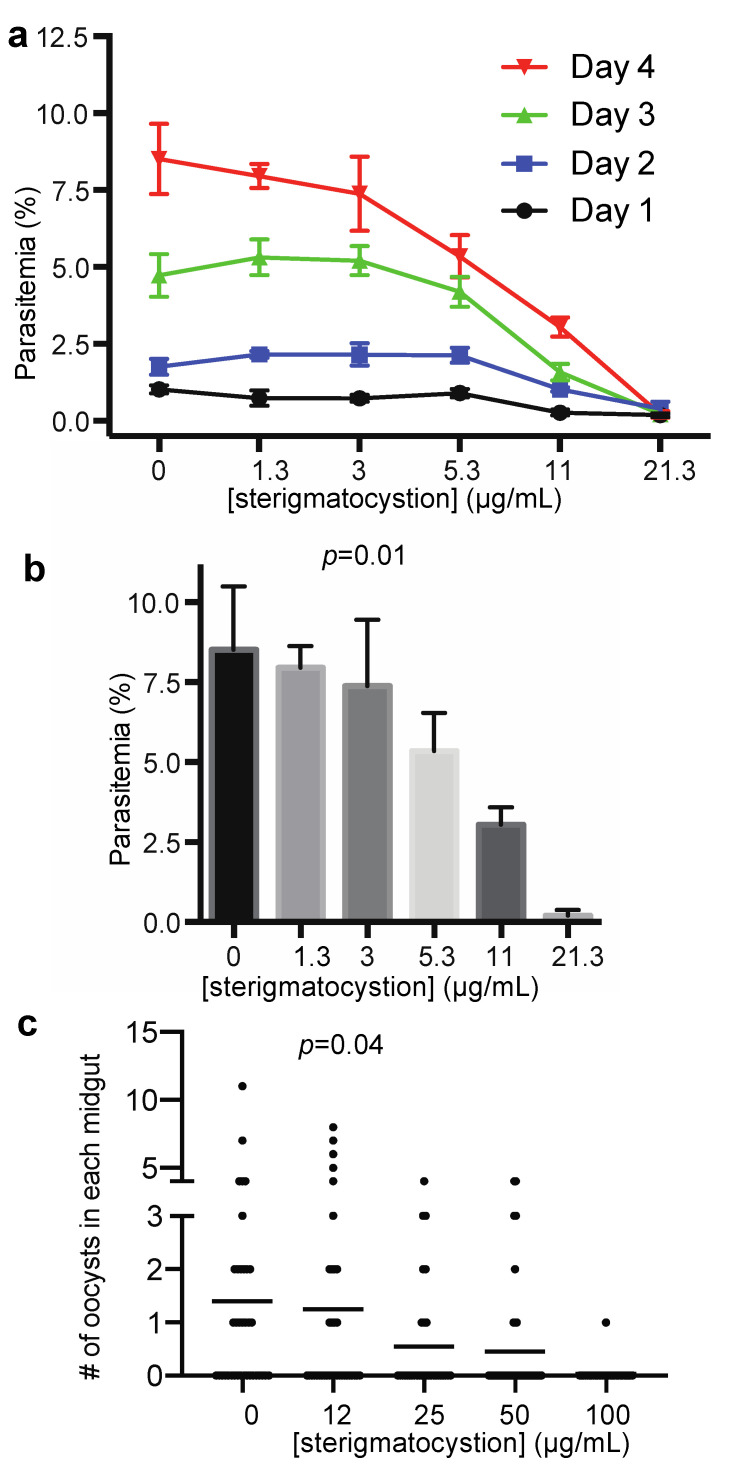
Sterigmatocystin inhibited *P. falciparum* infection in blood and transmission to *An. gambiae*. (**a**) Parasitemia at Day 1, 2, 3, and 4 after inoculation of different concentrations of compound. (**b**) Parasitemia on day 4 after incubated with different concentrations of compound. Significantly fewer *P. falciparum*-infected cells were observed when concentration of compound was greater than 11 µg/mL (*p* < 0.05), compared to control (0 μg/mL). For data in (**a**,**b**), each concentration was replicated three times; profiles show means and standard errors; (**c**) compound significantly inhibited *P. falciparum* transmission to mosquitoes at concentration of 25 µg/mL or more, compared to that of control (0 μg/mL).

We evaluated sterigmatocystin for its activity on the asexual stage of *P. falciparum*. The results demonstrated that inhibition of *P. falciparum* infection was dose-dependent (Figure 6a). The 11 μg/mL of the pure compound significantly reduced *P. falciparum* proliferation (*p* < 0.01) on day 4, and its IC_50_ was calculated as 11.3 μg/mL (34 µM) in inhibiting the asexual stage parasites (Figure 6b).

The sterigmatocystin against *P. falciparum* transmission to mosquitoes was also examined by SMFA. Different concentrations of sterigmatocystin in DMSO were added into blood containing 0.2% stage-V *P. falciparum* gametocytes and fed to mosquitoes. Results showed that when the final concentration of sterigmatocystin was greater than 25 μg/mL, there were significantly fewer oocysts in the compound-treated mosquito midguts compared to the control samples (*p* < 0.04) (Figure 6c). The EC_50_ was calculated to be 16 μg/mL or 48 μM.

### 2.4. The Cytotoxicity of Sterigmatocystin

**Figure 7:**
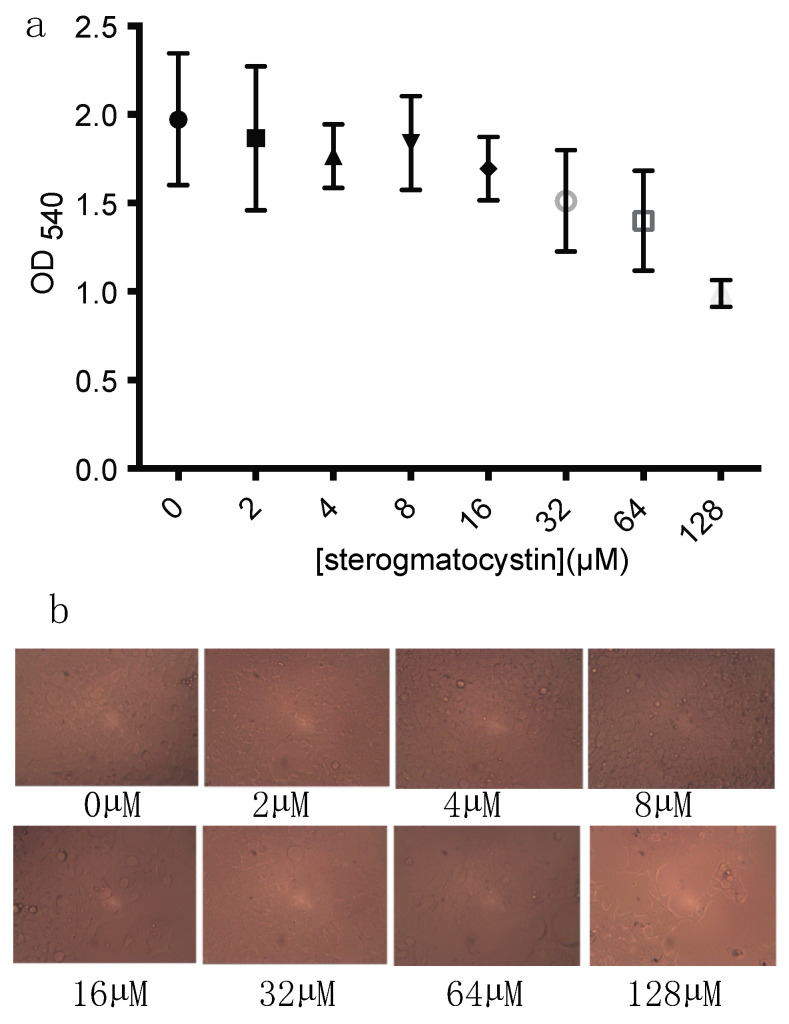
Cytotoxic effects of sterigmatocystin on human embryonic kidney 293 (HEK293) cell proliferation at varying concentrations were measured with MTT assays. (**a**) Compound did not show significant cytotoxicity to human embryonic kidney 293 cell line at a concentration of 64 µM (21.3 µg/mL) or lower; (**b**) density of living cells was significantly lower when Compound 1 reached 128 µM, compared to that of other concentrations (*p* < 0.01). Test for each concentration was replicated three times. Data were analyzed using a *t*-test.

Finally, we analyzed the general cytotoxicity of sterigmatocystin on the human embryonic kidney HEK293 cells. Firstly, the compound at varying concentrations (0–128 µM or 1–42.7 μg/mL) was mixed with HEK293 cells and incubated at 37 °C for 2 days. The cytotoxic effects of sterigmatocystin on cell proliferation were measured with MTT assays, and the cells’ morphology was also recorded under a light microscope in each treatment. The wells without the addition of the compound were used as a control. The results showed that sterigmatocystin did not show significant cytotoxicity to HEK 293 cell line at a concentration of 64 µM (21.3 μg/mL) or lower. The density of living cells was significantly lower when the concentration reached 128 µM, compared to that of the control (*p* < 0.01) (Figure 7a). Consistently, sterigmatocystin at 128 µM caused a significantly lower density of live cells under a light microscope (Figure 7b).

## 3. Discussion

In this study, we carried out the bioactivity-guided isolation and identification of an antimalarial compound from *P. janthinellum* by determining a sample’s bioactivity against the cultured *P. falciparum* asexual stage parasites and the sexual stage of parasites in mosquitoes using SMFA. The identified active compound was determined to be sterigmatocystin, which was first discovered in certain species of *Aspergillus* as a polyketide mycotoxin [16], and then reported in several phylogenetically and phenotypically different genera: *Aschersonia*, *Aspergillus*, *Bipolaris*, *Botryotrichum*, *Chaetomium*, *Emericella*, *Eurotium*, *Farrowia*, *Fusarium*, *Humicola*, *Moelleriella*, *Monocillium* and *Podospora* [17]. It has not been reported in the *Penicillium* genus.

Sterigmatocystin shows varying cytotoxicity to different cell lines but no acute toxicities. In this report, sterigmatocystin at 128 µM significantly inhibited the proliferation of HEK293 cells, and its inhibition was also confirmed under a light microscope. These data are consistent with the reported ranges from 3 μM to 286 μM using different detection methods and incubation times [18]. When the MTT assays were applied, the IC_50_ of >50 μM were obtained after 24, 48, and 72 h of exposure [19]. When another liver cancer cell, Hep3B cells, were used, the IC_50_ of 58 μM and 22 μM were obtained after 24 h and 48 h [20], respectively.

Here, we reported that 16 μg/mL or 48 μM of sterigmatocystin significantly reduced *P. falciparum* infection in the blood (*p* < 0.05), and its IC_50_ at day 4 was calculated to be 34 µM. We also found that IC_50_ of sterigmatocystin against *P. falciparum* transmission was about 16 μg/mL or 48 μM. These inhibition concentrations against sexual or asexual stage parasites are slightly lower than its cytotoxic concentration to human cells, suggesting that sterigmatocystin might play its function through general cytotoxicity. Sterigmatocystin consists of a xanthone nucleus attached to a bifuran structure, which is able to form DNA adducts [21]. Therefore, the antimalaria activity of sterigmatocystin has a different mechanism from other antimalaria compounds, explaining the *Penicillium janthinellum* extract against chloroquine-resistant *P. falciparum* INDO strain [12].

A sterigmatocystin derivative, oxisterigmatocystin E isolated from the fungus *Botryotrichum piluliferum*, also displayed antimalarial activity toward *P. falciparum* with an IC_50_ of 7.9 μM [22]. In addition, previous research found that sterigmatocystin isolated from a new endophytic fungus *Mycosphaerella* sp. possesses potent activity against another parasite, *Trypanosoma cruzi*, and obtained an IC_50_ value of 0.13 μM but did not observe the activity against *P. falciparum* at the 30 μM level [23].

Notably, 30 μg/mL crude extract from *P. janthinellum* completely blocked *P. falciparum* transmission to *An. gambiae* (Figure 3) and the crude extract contained about 0.06% sterigmatocystin (Figure 2). The activity of sterigmatocystin against parasites was much lower than expected. Therefore, the crude extract of *P. janthinellum* may still contain other anti-*Plasmodium* compounds, or some synergetic effects of multiple small molecules may occur.

## 4. Materials and Methods

### 4.1. Cloning and Sequencing ITS of a Fungal Species

The *P. janthinellum* fungus was from our constructed fungal library in 2020 (Fungal ID: 26F6) [13]. The fungal species was determined by the nuclear ribosomal internal transcribed spacer (ITS) region. The ITS region was PCR amplified with the primer set of ITS1F (5′-CTTGGTCATTTAGAGGAAGTAA-3′) and ITS4 (5′-TCCTCCGCTTATTGATATGC-3′) according to the following method: initial denaturation at 94 °C for 2 min, 35 cycles of denaturation at 94 °C for 30 s, annealing at 55 °C for 30 s, and extension at 72 °C for 1 min, followed by a final extension at 72 °C for 5 min. The PCR product was sequenced and compared to sequences in standard nucleotide database (nr/nt) by BLAST at ncbi.nlm.nih.gov (accessed on 8 October 2020) to determine the fungal species.

### 4.2. Preparation of Crude Fungal Extract

We followed the previously described method to grow fungi and to prepare the fungal extracts [10]. In brief, the fungus was firstly inoculated on malt extract agar medium (MEA) consisting of 5 grams (g) of malt extract, 0.5 g of yeast extract, 7.5 g of agar, and 0.025 g of chloramphenicol in 1 liter (L) distilled water, and a piece of the fungal culture on the agar plate was cut with a sterile loop and transferred to a conical flask containing 50 mL of the MEA liquid medium. The conical flask was incubated in an incubator shaker for growing fungal culture for 4 days at 25 °C. A sugar solution with 3 g of sugar and 50 mg of chloramphenicol was dissolved in 1 L of distilled water in conical flasks and autoclaved for 15 min. A mushroom bag filled with 550 g of autoclaved cheerios cereal (General Mills Cheerios Cereal) was further autoclaved for 30 min. The autoclaved sugar solution, cereal, and the 50 mL fungal culture in MEA medium were mixed in the mushroom bag, and the cultures were incubated for four weeks at 25 °C with 12 h day and 12 h dark cycles. Then, the fungal culture contents in the bag were transferred to a glass container, and 1 L of ethyl acetate was added. After 4 h with constant stirring, the fungal culture materials were broken into smaller pieces. The fungal materials were kept in the container for extraction for 24 h. The supernatant was filtered through a Buchner funnel lined with a filter paper. An additional 500 mL of ethyl acetate was added and kept for extraction for 4 h. The ethyl acetate extract was dried using a rotary vacuum evaporator at 30 °C.

### 4.3. Isolation of Active Compounds

All solvents used for extraction and separation were purchased from Thermo Fisher Scientific (Waltham, MA, USA) unless otherwise stated. The dried crude extract (16 g) was firstly fractioned using vacuum liquid chromatography (sintered glass funnel G1 (90–150) packed with 200 g 230–400 mesh silica gel (particle size 40–63 µM) (Sorbent Technologies, Norcross, GA, USA)). A laboratory water aspirator pump was used to generate a 20–70 mmHg vacuum for suctioning. The ethyl acetate extract was dissolved in a small amount of methanol and spread onto the vacuum liquid chromatography (VLC) column to form a uniform band. Elution of the VLC column was carried out using gradient elution. A total of six major fractions were collected with 600 mL of each of following solvents: Hexane (F1), 1:1 (Hexane:ethyl acetate, *v*/*v*, F2), 100% dichloromethane (DCM) (F3), 95:5 (DCM:Methanol, F4), 80:20 (DCM:Methanol, F5), 100% Methanol (MeOH) (F6).

Since fractions F5 (2.4055 g) showed activity against malaria at the asexual and mosquito stages, it was further chromatographed over gravity column, using 24/40 glass chromatography column (450 × 15 mm) packed with 60–100 mesh silica gel (150 Å) and eluted five sub-fractions the following solvents: 95:5 (DCM:MeOH, SF-1), 90:10 (DCM:MeOH, SF-2), 85:15 (DCM:MeOH, SF-3), 80:20 (DCM:MeOH, SF-4) and 100% MeOH (SF-5). The volume of each fraction was about 3 times of the silica gel volume (80 mL). The active SF-1 was further purified on semipreparative HPLC with the following steps. SF-1 (5%MeOH in DCM) was dissolved in methanol and passed through the C18 sample preparation column (Sep Pak Cartridge, Waters, Milford, MA, USA) to remove the insoluble materials. The collected fraction was dried over vacuum and dissolved in methanol and water (containing 0.1% formic acid) in a 50:50 ratio. A semipreparative reverse phase HPLC (Shimadzu LC-20AD pump, SPD-M20A detector, and FRC-10A fraction collector, Shimadzu, Columbia, MD, USA) with a semi-preparative column (Gemini C18 250 × 10 mm, 5 μm, Phenomenex, Torrance, CA, USA) was carried out with a flow rate of 5 mL/min by using a gradient solvent of MeOH/H_2_O (containing 0.1% Formic acid) from 50% MeOH to 100% MeOH.

The purity of the compound was further analyzed by HPLC C18 column (Gemini C18 250 × 10 mm, 5 μm, Phenomenex) with a flow rate of 5 mL/min by 70% acetonitrile in water. The absorbance of elution was detected with UV-Vis (200 to 800 nm) PDA detector (SPD-M20A detector).

### 4.4. High-Resolution Mass Spectrum

High-resolution mass spectrometry was recorded using (+) ESI mode on the Bruker Daltonics, Impact II QTOF mass spectrometer (Gas temperature −200 °C, Drying gas (N2)—4 L/m Nebulizer—0.3 Bar, Bruker Scientific LLC, Billerica, MA, USA). High-resolution mass spectrometry analyses were conducted at the University of Florida Mass Spectrometry Research and Education Center, Department of Chemistry.

### 4.5. Nuclear Magnetic Resonance (NMR) Analysis

The 300 MHz ^1^H and 75 MHz ^13^C NMR spectra were recorded on Bruker 300 spectrometer. The chemical shifts are presented in terms of ppm with tetramethylsilane (TMS) as the internal reference, and J values were reported in Hertz. Carbon atom types (C, CH, CH_2_, CH_3_) were determined with a DEPT pulse sequence.

### 4.6. Asexual Plasmodium Falciparum Growth Inhibition Assays

Asexual stage *P. falciparum* parasites (NF54 strain from MR4, Manassas, VA, USA) were maintained in RPMI-1640 medium (Life Tech, Grand Island, NY, USA) supplemented with 10% heat-inactivated (56 °C for 45 min) human AB^+^ serum (Interstate blood bank, Memphis, TN, USA), 12.5 μg/mL hypoxanthine, and 4% hematocrit (O^+^ human blood) in a candle jar at 37 °C. *P. falciparum*-infected human red blood cells were mixed with new AB+ type uninfected human RBCs to prepare cultures with 0.5% parasitemia and 2% hematocrit. The fractions were dissolved in DMSO, and 1 µL of small molecule solution (6 mg/mL) was added into 200 µg/mL of culture per well in a 96-well plate. The plate was incubated in a candle jar at 37 °C. The medium, as well as the candidate compound, was replaced at 48 h. The parasitemia was recorded at 24 h, 48 h, 72 h, and 96 h after incubation. The assays were run in triplets and independently conducted twice. IC_50_ was determined by analysis of the dose-response curve made by GraphPad Prism (GraphPad Software, San Diego, CA, USA). *p*-values were calculated with a *t*-test.

### 4.7. Analyzing the Effect of Active Compounds on P. falciparum Infection in Anopheles Gambiae

The 15–17-day cultured *P. falciparum* iRBC containing 2–3% gametocytes at stage V were collected and diluted with fresh O+ type human blood that was mixed with the same volume of heat-inactivated AB+ human serum. The final concentration of the stage V gametocytes in the blood was around 0.2%. The compounds in DMSO were added into the infected blood (2:300 by volume), and then a SMFA was performed to feed ~80 3-day old female mosquitoes for 30 min, and the engorged mosquitoes were maintained with 8% sugar in a BSL-2 insectary (28 °C, 12-h light/dark cycle, 80% humidity). The midguts were dissected 7 days post-infection and stained with 0.1% mercury dibromofluorescein disodium salt in PBS. The oocysts were counted under a light microscope. All the experiments were independently repeated at least twice. The Wilcoxon–Mann–Whitney test was used to calculate the *p*-value of infection difference between the control and experimental groups.

### 4.8. Cytotoxic Assay

The MTT (3-(4,5-dimethylthiazol-2-yl)-2,5-diphenyl tetrazolium bromide) cell proliferation assay (Thermo Fisher Scientific) was used to measure the general cytotoxicity of pure compounds to the human embryonic kidney 293 (HEK293) cell line (ATCC accession number CRL-1573). The HEK293 cells at a concentration of 2 × 10^5^ cells/well in 100 µL culture medium containing different concentrations of the pure compounds were seeded into microplates and incubated for 24 h at 37 °C and 5% CO_2_. Next, the cells were incubated with 100 µL of MTT reagent for 4 h at 37 °C (final concentration 0.5 mg/mL). After removing all but 25 µL of the medium from the wells, 100 µL of DMSO was added to each well and incubated for 10 min to dissolve the purple formazan crystal for measurement. The optical absorbance at wavelength of 540 nm was measured with a microplate reader.

## 5. Conclusions

This study reported *Penicillium janthinellum* extract that inhibited asexual *P. falciparum* proliferation and sexual stage parasites transmission to *An. gambiae*. An active compound was then isolated and determined to be sterigmatocystin. Sterigmatocystin consists of a xanthone nucleus attached to a bifuran structure, which is able to form DNA adducts. Therefore, the activity of sterigmatocystin against *P. falciparum* may be caused by the general cytotoxicity. While more antimalarial compounds are expected to be discovered from *P. janthinellum,* sterigmatocystin is antimalarial drug lead and can be used as a probe to investigate molecular mechanisms of the parasite life cycle.

## Figures and Tables

**Table 1:** Inhibition activities of fractions on *P. falciparum* asexual stage proliferation.

Samples	The Inhibitory Ratio of the Growth of Asexual Stage of *P. falciparum* (%) (30 µg/mL)
Crude extract	100.0
F1 (100% Hexane)	31.5
F2 (50:50 Hexane:EtOAc)	60.6
F3 (100% DCM)	93.1
F4 (95:5 DCM:MeOH)	95.0
F5 (80:20 DCM:MeOH)	100.0
F6 (100% MeOH)	38.1
SF-1 (5% MeOH)	100.0
SF-2 (10% MeOH)	95.7
SF-3 (15% MeOH)	77.9
SF-4 (20% MeOH)	82.1
SF-5 (100% MeOH)	80.7

Note: EtOAc (Ethyl acetate); DCM (dichloromethane); MeOH (methanol). Inhibitory rate was calculated by equation.

**Table 2:** ^1^H NMR and ^13^C NMR data of candidate (*δ* in ppm, *J* in Hz, and NMR solvent Chloroform).

Position	*δ*_C_	*δ*_H_ (*J* in Hz)	Structure
1	181.6, C		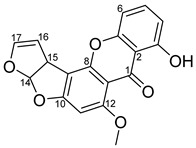
2	109.1, C		
3	162.5, C		
4	113.4, CH	6.84, d (7.3)	
5	135.9, CH	7.51, t (8.5)	
6	106.7, CH	6.84, d (7.3)	
7	155.1, C		
8	154.2, C		
9	106.5, C		
10	164.7, C		
11	90.7, CH	6.44, s	
12	163.5, C		
13	106.5, C		
14	111.4, CH	6.76, dd (0.9, 8.4)	
15	48.2, CH	4.82, dt (7.2, 2.3)	
16	102.7, CH	5.45, t (2.5)	
17	145.5, CH	6.51, m	
OCH_3_	57.0, CH_3_	4.00, s	

## Data Availability

All data used in this study are available at public databases or in this document. The ITS sequence had been submitted to the GenBank with the Accession # of GU565141.1.
